# Perceived risk, symptoms and help-seeking behaviour for obstructive sleep apnoea among undergraduate medical students: a qualitative study

**DOI:** 10.3389/fpubh.2026.1857585

**Published:** 2026-06-25

**Authors:** Dharani Bhaskaran, Suba Angappan, Suma Sukumaran, Abeetha Sai Subramanian, Chitra Mourali, Sudha Durairaj

**Affiliations:** 1 Department of Physiology, A.C.S. Medical College and Hospital, Dr. M.G.R. Educational and Research Institute, Chennai, Tamil Nadu, India; 2 Department of Physiology, Saveetha Medical College and Hospital, Saveetha University, Saveetha Institute of Medical and Technical Sciences (SIMATS), Thandalam, Chennai, Tamil Nadu, India; 3 Department of Physiology, Sree Balaji Medical College and Hospital, Bharath Institute of Higher Education and Research, Chennai, Tamil Nadu, India

**Keywords:** medical students, obstructive sleep apnoea, public health, sleep health, qualitative research, young adults

## Abstract

**Background:**

Obstructive sleep apnoea (OSA) is a well-known type of sleep disorder that is commonly underdiagnosed. Undergraduate medical students are said to be a paradoxical group because despite their theoretical knowledge about symptoms of OSA, they often omit their own symptoms of OSA. This may lead to delayed recognition of symptoms and identification of OSA. Sparse evidence exists regarding how they recognize their own OSA symptoms, interpret their risk and address their condition. The current study aims to explore the self-perceived risk of OSA, awareness of symptoms and help-seeking actions related to OSA among undergraduate medical students.

**Methods:**

A qualitative research design using purposive sampling was conducted among 36 undergraduate medical students from a medical college. Data were analysed using reflexive thematic analysis.

**Results:**

The results underscore the complex interplay between normalization of sleep symptoms, limited OSA awareness, reluctance to help-seeking and the prominent impact of medical college life on lifestyle. Four major themes were identified: limited awareness and misconceptions about OSA, normalization of symptoms, barriers to help-seeking behaviour, and the burden of medical school lifestyle on sleep health. The analysis revealed that only a few students demonstrated accurate knowledge of OSA. The majority of the students reported symptoms such as lifestyle changes and daytime sleepiness related to academic burden, yet help-seeking behaviour was low, with the majority opting for self-directed changes such as increasing exercise and adjusting their sleep schedules.

**Conclusion:**

In conclusion, this study reveals an alarming mismatch that exists among awareness of OSA, self-experience of symptoms and help-seeking behaviour among medical students. Hence, recognizing OSA as a potential personal health concern may represent an important step toward improving risk perception and encouraging timely help-seeking behaviour among future healthcare professionals.

## 1 Introduction

Obstructive sleep apnoea (OSA) is a well-known type of sleep disorder that is commonly underdiagnosed with repeated episodes of apnoea during sleep due to upper airway obstruction. This results in symptoms such as sleep fragmentation, intermittent hypoxia and daytime somnolence (1). Previously, OSA was thought to be prevalent only among obese, middle-aged individuals. However, recent research has shown that its prevalence is increasing among younger people such as college students, with pooled prevalence rate of around 16% (2). This is because they have poor lifestyles, such as sedentary behaviour, poor diet, poor sleep and heightened academic stress (3). Studies have found that about 80–85% of clinically significant OSA cases remain undiagnosed among young adults (4).

Undergraduate medical students are said to constitute a paradoxical group. This is because despite their theoretical knowledge about symptoms of OSA, such as unrefreshing sleep, impaired concentration, snoring and daytime fatigue, they often omit their own symptoms of OSA as stress-related, leading them to self-neglect. This puts them at an increased risk of developing OSA (5). Various factors have been identified as causes of underdiagnosis and undertreatment for individuals with OSA. These include a paucity of awareness, diagnosis, and management, including reluctance to ask for help, normalization of sleep deprivation and fear of judgement (6). This results in large number of people being underdiagnosed and undertreated for OSA, putting them at risk of developing complications due to OSA. The health behaviours of medical students are highly important, as they are the future physicians and if they practice good self-care, they are more likely to understand health risks in themselves and could effectively promote preventive health behaviours among their patients.

Previous studies have underscored the significance of self-stigma, perceived invulnerability and academic load in preventing the undergraduate medical students from admitting their health issues, including sleep disturbances and poor mental health (7). Nevertheless, little evidence exists regarding how undergraduate medical students recognize their own OSA symptoms, interpret their risk and address their condition. Rectifying this present gap between personal health behaviour and knowledge is crucial for improving the knowledge of wellness as well as preparing future physicians to understand and manage sleep disorders in themselves. The findings from the current study might be conceptually interpreted via the Health Belief Model (HBM), which describes the health-associated behaviours based on perceived severity, perceived susceptibility, perceived barriers, perceived benefits, self-efficacy, and cues to action. With regard to OSA among medical students, the HBM offers a potential framework to know why individuals might fail to recognize symptoms, normalize sleep-associated disturbances, underestimate personal vulnerability, and prevent professional help-seeking (8). Applying this framework might help describe the psychological and behavioural processes behind interpretation of symptoms and health-seeking decisions among medical students.

The current study aims to explore the self-perceived risk of OSA, awareness of symptoms and help-seeking actions related to OSA among undergraduate medical students. By examining their narratives, we try to uncover the potential barriers and perspectives that could lead to under recognition and self-neglect of possible OSA symptoms within this medically educated yet vulnerable population.

## 2 Methodology

### 2.1 Study design

The current study uses a qualitative research design to explore the symptoms, self-perceived risk and help-seeking behaviour concerning OSA among undergraduate medical students. The study used an interpretive qualitative approach to know how undergraduate medical students perceive and interpret sleep-associated symptoms within the context of irregular sleep schedules, rigorous academic environments, and emerging health behaviours.

A qualitative research approach was adopted to understand in-depth students’ perceptions, experiences, and their behavioural factors that might not be explored through quantitative methods. Data collected were analysed using reflexive thematic analysis to identify patterns and create themes using participants narratives. Instead of using a pre-defined behavioural hypothesis, the current study sought to explore the real-life experiences and narratives of medical students to clearly understand how perceived vulnerability, awareness, and behavioural responses toward probable OSA symptoms are structured.

### 2.2 Study setting and duration

After approval from Institutional Ethics Committee, Undergraduate medical students were selected for this study from a tertiary medical college in Chennai, India. The study was conducted for a duration of 6 months in the year 2025. The inclusion criterion consisted of undergraduate medical students who were at least 18 years of age. The academic environment of undergraduate medical training offers a unique context that makes the medical students a highly relevant population for determining how sleep disorders interact with perceptions of risk and personal health behaviours.

### 2.3 Study population and sampling strategy

The study population comprised first-year undergraduate medical students aged 18 years and above who were enrolled in the chosen medical college and were willing to share their experiences within the study context. Those students who were already diagnosed and were undergoing treatment for OSA were excluded. The study participants were recruited using purposive sampling to obtain varied perspectives regarding sleep experiences, awareness of OSA symptoms, perceived risk, and help-seeking behaviour among first-year undergraduate medical students. Both male and female students who were willing to participate were included. The recruitment process was continued until the data saturation was reached, that is represented as the point at which no new themes were found from consequent interviews. The selection was performed in such a way that different experiences and knowledge regarding the risk of OSA were ensured. Thematic saturation was assessed jointly by the research team during ongoing data analysis and was considered achieved after 36 interviews, when no substantially new codes or themes emerged across consecutive interviews.

### 2.4 Ethical considerations

The current study was performed after obtaining clearance from the Institutional Ethics Committee of ACS Medical College and Hospital, Dr. M.G.R. Educational and Research Institute, Chennai, India (Approval No: 562/2022/IEC/ACSMCH; approved on 6 October 2022) and adhered to its guidelines. Informed written consent was obtained from each of the participants to ensure that they were fully aware of the aim and objectives of the present study, to determine their voluntary nature for participation and to inform their right to withdraw from the study at any point. All the study participants were completely assured about the anonymity and confidentiality of their responses. The study participants were informed that anonymised excerpts from their interviews may be utilised in academic publications. All the data obtained were stored safely in password-protected digital systems, and only the research team of the study had complete access to the audio recordings of the interviews and their transcriptions.

The study was reported in accordance with the Standards for Reporting Qualitative Research (SRQR) to assure completeness and transparency in context of qualitative methodological reporting.

### 2.5 Data collection

Data collection was performed through in-depth semi-structured interviews by the principal investigator, which helped explore the experiences and views of the participants flexibly.

A guide for interviews was developed based on the existing literature to investigate three key themes;

1. Recognition of OSA symptoms: The selected participants were asked about their awareness and appreciation of their symptoms related to OSA such as excessive daytime sleepiness, snoring, and fatigue.
2. Self-perceived risk of developing OSA: Participants were questioned about their knowledge and self-perception of their risk of OSA developing OSA.
3. Help-seeking behaviour: The participants were asked about their help-seeking behaviour on sleep -related issues, such as whether they had asked for help and, if not, what reasons and barriers prevented them from seeking help.

The semi-structured interview guide used for data collection has been provided as Supplementary material 1. All the interviews were performed individually to assure comfort and privacy. Neutral and non-judgmental questioning was used throughout the interviews to encourage honest responses and reduce response-related bias. Each of the focused interviews lasted for approximately 30 to 45 minutes and were completely audio-recorded after obtaining informed written consent and subsequently transcribed verbatim for analysis. In addition to interviews, field notes were also maintained to document observations and nonverbal clues during the individual interviews.

### 2.6 Data analysis

All the data collected were transcribed word-for-word and scrutinized via thematic analysis. Two independent researchers conducted the coding process to improve the analytical rigor. Subsequently, each transcript was coded independently, and any discrepancies in coding were clarified via discussion till a final consensus was achieved. Attention was also given to identifying responses that differed from dominant patterns during theme refinement.

The transcripts were subsequently recurrently read to become familiar with the data and achieve immersion in the data. Codes were subsequently generated inferentially, and themes were developed based on repeated responses and patterns associated with three core areas: self-perception of risk, symptom awareness and help-seeking behaviour. Similar codes were grouped into categories before being refined into broader themes through iterative discussion among the research team.

Thematic analysis was performed via the following steps:

1. Familiarization with the data: Reading the entire transcripts and making initial notes.
2. Generating codes: Coding was performed using the data on self-perceived risk, symptoms of OSA and help-seeking behaviour.
3. Identifying specific themes: The codes were grouped under broader themes to represent the key concepts.
4. Reviewing themes: The identified themes were ensured for accurate reflection of the data.
5. Naming and defining themes: Confirming the themes and creating definitions.

NVivo qualitative data analysis software was used to support transcript organization, coding, and retrieval of thematic patterns during analysis. Formal intercoder agreement was not statistically calculated; discrepancies in coding were resolved through iterative discussion and consensus between the researchers. An example illustrating the coding and theme development process has been provided as Supplementary material 2. While the analytical strategy was basically inductive, developing themes were consequently interpreted in context of constructs from the Health Belief Model, encompassing perceived susceptibility, perceived barriers to seeking care, and perceived severity. This conceptual basis aids contextualise how undergraduate medical students interpret potential OSA symptoms and make decisions in context of health-seeking behaviour.

### 2.7 Researcher reflexivity

Based on the interpretative nature of qualitative study, reflexivity was integrated throughout the research process. The principal investigator has professional exposure to sleep-based health discussions and preventive counselling. During the process of data collection and analysis, awareness of this background was preserved to reduce the impact of biomedical assumptions on the narrative’s interpretation.

Reflexive memo-writing was pursued during interviews and throughout the analytical process to note arising interpretations, potential responses, and emotional responses. This process supported to differentiate between output obtained directly from experiences of participants and interpretations emerging from the researcher’s clinical perspective.

### 2.8 Trustworthiness and rigor

To improve the reliability and credibility of the findings, the current study used member checking in which a short summary of the derived key findings was shown to participants for proper validation. Furthermore, peer feedback was obtained from colleagues in the sleep medicine field to provide an external perspective on the analysis process. Interview transcripts and field notes were regularly reviewed to assure that the developing themes remained based on participant narratives to support confirmability. Furthermore, regular documentation of coding procedures and analytical decisions were preserved throughout that process of data analysis to maintain dependability. Also, detailed explanation of the participant characteristics and study context were provided to support analysis of transferability to similar populations. These strategies were employed to improve methodological rigor and transparency in the interpretation of the qualitative data.

## 3 Results

The results were obtained by analysing the qualitative data collected from 36 undergraduate medical students with the help of thematic analysis to investigate their experiences, perceptions and attitudes toward OSA. All participants were first-year undergraduate medical students and included both male and female students. Four key themes were derived from the thematic analysis: (1) Misconceptions and limited awareness about OSA, (2) Symptom recognition and normalization, (3) barriers to seeking help, and (4) Impact of medical school lifestyle. The derived findings are represented below, supported by illustrative participant quotations.

### 3.1 Thematic analysis

#### 3.1.1 Misconceptions and limited awareness about OSA

The participants revealed different levels of knowledge regarding OSA, including accurate descriptions of OSA and a total lack of awareness. Many students described OSA precisely, whereas few stated that they did not have any knowledge of OSA. Also, they shared incomplete or partial knowledge about OSA.

For example, one of the study participants reported:

“*Obstructive sleep apnoea (OSA) is a condition where the airway repeatedly becomes blocked during sleep, causing breathing to stop and start,” whereas another stated, “I do not know about sleep apnoea.*”

Additionally, a large proportion of participants held the perception of not being at risk of OSA because of an incorrect belief in its relevance among young adults and a lack of symptom awareness.

One participant stated:

“*I think it is an issue faced by a lot and I do not think I am at risk.*”

This indicates that personal risk is often underestimated despite being a medical student. These narratives show that theoretical exposure to OSA knowledge potentially translate into personal risk appraisal. Many participants were found to psychologically distance themselves from the possibility of disease, often relating OSA with obese or older individuals instead of considering their susceptibility within their own age group.

#### 3.1.2 Symptom recognition and normalization

The study participants frequently reported symptoms related to poor sleep and most of the participants reported having at least one symptom, such as restlessness, snoring, or pauses in breathing while sleeping.

For example, one of the participants stated:

*“Breathing problems and restlessness during sleep are noticed by me.”*

However, these symptoms were mostly accepted and pointed to non-medical reasons such as irregular sleep schedules, academic stress or heavy meals.

One participant explained:

*“Every day and every single hour I often feel sleepy, this is due to irregular sleep habits and sometimes improper diets.”*

This normalization of symptoms was observed in most of the total participants who reported excess daytime sleepiness, with many imputing disruptions in their sleep schedules. The tendency to normalize symptoms within the point of academic stress denotes how chronic sleep disruption becomes accepted culturally within medical training environments. This appeared to decrease the likelihood of interpreting symptoms as clinically significant.

#### 3.1.3 Barriers to seeking help

The help-seeking behaviour for issues related to sleep was observed to be minimal, with few participants expressing a mindset toward help-seeking. However, a minimal proportion of participants reported no need for any intervention. The participants stated that they preferred self-management approaches such as increasing exercise or adjusting their sleep schedules rather than receiving medical consultations.

One participant explained:

*“I am trying to change my sleep schedule and it’s working.”*

This shows that the participants seek reliability of self-directed solutions. Other participants explained help-seeking as infeasible or unnecessary as a part of heavy academic work, with one participant stated:

*“I think it would work if I get proper assistance, but it will be difficult to implement with schedule consistency. Being a medical student, it sounds impossible for me to get sleep correctly.”*

Participants often perceived behavioural self-adjustment as more practical than professional consultation, reflecting decreased perceived necessity for medical evaluation and potential reduction of symptom severity.

#### 3.1.4 Impact of medical school lifestyle

The demanding academic and medical school environment has drastically influenced students sleep patterns, lifestyles, and health. The study participants often reported that irregular schedules and academic stress were the primary contributing factors to sleep disruption and was pointed to academic stress, and few participants related excess daytime sleepiness to sleep schedules-associated issues. Consistent with this, one of the participants stated:

*“I feel sleepy during the day quite often, especially after lunch or during long lectures. I think it is mostly due to staying up late and inconsistent sleep patterns.”*

Majority of the participants reported lifestyle changes due to joining medical school, which were attributed to academic stress, health reasons, and social influences. Few participants reported positive changes such as increased exercise and adjustments to the sleep schedule. However, some stated negative changes such as irregular sleep patterns and dependency on fast, convenient meals. One participant stated:

*“I normally sleep a lot. However, since I am trying to stick on to the schedule, I am having reduced sleep which has caused drastic weight loss of more than 5 kg.”*

This emphasizes the health outcomes of heavy academic demands. Academic pressure played not only as a contributing factor to poor sleep quality but also as a contextual justification for symptom neglect and reinforcing maladaptive sleep behaviours as a normalized factor of medical training. The thematic analysis is represented in Table 1.

**Table 1:** Thematic analysis.

Theme	Subtheme	Description	Representative quote
Misconceptions and limited awareness about OSA	Varying knowledge about OSA	Participants revealed different levels of knowledge regarding OSA, covering accurate descriptions about OSA to total lack of awareness	*Obstructive sleep apnea (OSA) is a condition where the airway repeatedly becomes blocked during sleep, causing breathing to stop and start.* *It’s a disorder where airway get blocked while sleeping.* *I know that obstructive sleep apnea involves repeated blockage of the airway during sleep, causing breathing pauses and poor sleep quality.* *According to me it occurs due to obesity and sleeping position.* *I do not know about sleep apnea.*
	Low self-perceived risk	Large proportion of participants held the perception of not at risk of OSA, because of wrong belief of its relevance among young adults and lack of symptom awareness.	*I think it is an issue faced by a lot and I do not think I am at risk.* *According to me it is because of improper sleeping time and position, no I do not think that I am at the risk of sleep apnea.* *I do not think what it is but I am do not think I’m affected because of it.*
Symptom recognition and normalization	Recognition of symptoms	Students stated several sleep-related symptoms like restlessness, snoring or excess daytime sleepiness and they have mentioned that they are often noticed by others.	*I occasionally feel excessively sleepy during the day, especially after nights when I have not slept enough or had interrupted sleep. I usually attribute it to staying up late studying, stress or using screens too close to bedtime. On those days, it’s hard to concentrate in lectures.* *I feel sleepy during the day quite often, especially after lunch or during long lectures. I think it’s mostly due to staying up late and inconsistent sleep patterns.*
	Normalization of symptoms	The sleep-related symptoms were linked to irregular sleep schedule, academic stress or heavy meals, instead of underlying medical conditions.	*I usually feel sleep after our lunch break. I think it may be due to heavy meals.* *I feel sleepy right after eating lunch fully … and when they teach continuously like blah blah blah blah… I lose focus then I feel sleepy.* *I feel often sleepy during the day because of not sleeping properly at night.* *Often feel excessively sleepy during the day mostly due to lack of sleep the previous day.* *I usually feel very sleepy like at morning like at 11am and this is due to my irregular sleep schedule at night.* *Every day and every single hour I often feel sleepy, this is due to irregular sleep habit and sometimes improper diet.* *Due to depression, academic stress and inadequate sleep cause feel sleepy during day time.*
Barriers to Help-Seeking	Prefer to do self-management	Students have opted for self-management approaches rather than getting medical advice.	*The blue light needs to restricted on 8:00 pm as that would make us sleep in 10:00.* *I am changing my sleep schedule and it’s working.* *I think seeking help can be great if we cannot manage sleep by own or have disorders related to sleep.* *But I do not think I need them cuz I am changing my sleep schedule and it’s working.*
	Perception of lack of necessity and feasibility	Some participants had the perception of that the sleep disturbances are not severe enough or doubtful on making the changes feasible in medical school.	*It would work if I got help but I do not know if I can keep with that schedule consistency… while being in medical school like that sounds impossible so sleep correctly on time …* *I have not thought about seeking help on sleep related issues.*
Impact of Medical School Lifestyle	Academic burden and poor sleep	Academic burden induced improper scheduled, leading to sleepiness and fatigue in context of disrupted sleep.	*Exams, assignments, stress in general or something that causes emotional distress or scares me disturbs my sleep cycle.* *My sleep is disturbed when i have to stay up late to finish my work and studies. Also, if i stress about my exams and career it greatly affects my sleep.* *The fear of losing, the fear of not winning in life, the fear of not becoming successful, the fear of not becoming the best version of myself, the fear of not becoming rich as young as possible, the fear of not making my parents proud.* *I often feel excessively sleepy during the day, especially in the afternoon and I attribute it to staying up late at night, poor sleep quality and a disturbed sleep routine caused by academic stress and long screen time.*
	Lifestyle changes and wellbeing	Both negative (e.g., junk food, poor sleep) and positive (e.g., exercise) lifestyle changes were reported that has impact on health and sleep	*I have lost more than 5 kg, because I am a person who usually sleeps a lot and having to stick with schedule that reduces my sleep has affected my weight, drastically.* *Joined gym just to be healthy and strong for longer lab time and travel.* *Change in my sleeping patterns, change in food habits and timings. My daily schedule made me change them.*

## 4 Discussion

This qualitative study explored the self-perceived risk of OSA, recognition of OSA symptoms and help-seeking behaviours related to OSA. This underscores the critical gap between the knowledge of OSA and appropriate health-seeking behaviour. These patterns observed from the present study can be conceptually understood via constructs of the Health Belief Model, especially perceived susceptibility, severity, and barriers affecting health-related decision-making. Specifically, many participants reflected low perceived susceptibility by thinking that OSA primarily affects older individuals. Additionally, normalization of poor sleep and daytime sleepiness within medical training appeared to decrease perceived symptoms severity and delay health-seeking behaviour.

Despite having a medical education background, medical students have reported a surprisingly greater degree of misconception and symptom normalization and have exhibited hesitation toward seeking medical help. Similar gaps observed in previous studies between awareness and health-seeking behaviour among medical trainees and young adults in sleep health research, reflecting that medical knowledge alone might not translate into adequate risk perception or help-seeking behaviour.

### 4.1 Limited awareness and misconceptions regarding OSA

The most striking result from the current study was the limited knowledge about OSA. Only a small proportion could explain OSA clearly, and a significant proportion had no understanding or had partial understanding of OSA. This is of real concern, as this population has a medical background and will play a future role in the early identification and intervention of OSA. Furthermore, majority of the participants had self-perceptions of not being at their own risk, which was identified due to misconception. This finding aligns with the results of previous studies, which have shown that young adults and even medical trainees often fail to identify themselves as at risk of sleep-related disorders even though they present with significant symptoms (9–11). This observation from the present study denotes low perceived susceptibility, a central construct of the Health Belief Model, where individuals underestimated their own risk despite manifesting symptoms related to a health condition.

### 4.2 Symptom recognition and normalization

Even though many participants reported red flag symptoms of OSA, they could not identify them. Instead, they mentioned these symptoms as part of their irregular sleep habits, academic burdens, and diet. This tendency to normalize symptoms was found in most of the students with specific reports of excess daytime sleepiness, and most of them linked it with their disrupted routine instead of pathological reasons. These results resonate with findings from previous research that normalization of sleep-related symptoms in young adults could lead to delays in the recognition and management of conditions such as OSA (6, 12). Within the known framework of the Health Belief Model, this normalization of symptoms might represent decreased perceived severity, where individuals interpret possibly concerning symptoms as regular consequences of lifestyle instead of indicators of an underlying health disorder.

### 4.3 Barriers to help-seeking and preferences for self-management

A general reluctance to seek medical help for problems related to sleep prevails among medical students. Furthermore, many have shown interest in adopting self-management strategies such as increasing physical activity and modifying sleep schedules instead of professional help. Additionally, some doubted the feasibility of adopting changes in view of the academic schedule. This is in accordance with previous studies highlighting that adopting structured sleep hygiene in medical school life is difficult which reflects various cultural issues in medical education, where sleep deprivation and exhaustion are generally normalized (13). These observations from the current study suggest the presence of perceived barriers, another critical construct of the Health Belief Model, where factor like time constraints, academic load, and normalization of sleep deprivation might prevent medical students from seeking professional assessment.

### 4.4 Burden of medical school lifestyle

The present study revealed that students attributed the reasons behind deteriorating sleep patterns, such as erratic schedules, academic pressure and emotional stress. Interestingly, several participants reported significant lifestyle modifications due to medical school, which included both negative (e.g., irregular sleep, poor diet) and positive (e.g., increased exercise) aspects. These findings are in accordance with the literature showing that medical school training prominently affects health behaviours and sleep–wake patterns, often leading to impaired cognitive function and burnout (14, 15).

The findings from the present study emphasize the need for enhanced sleep health education within medical curricula. Improving awareness about OSA symptoms and perceptions of risk among medical students enhance earlier recognition and facilitate timely help-seeking behaviour. The current study findings might also denote the wider culture within medical training, where fatigue, sleep deprivation, and irregular sleep schedules are often normalized as expected factors of academic life. Such normalization could decrease symptom recognition and discourage help-seeking behaviour even among medically informed individuals. Constant exhaustion and academic pressure might additionally contribute to emotional burnout, which might further influence self-care practices and sleep health among medical students.

The current study has few limitations despite findings. This study was conducted among undergraduate medical students from a single medical college, which could limit transferability of study findings to other student populations. Also, the qualitative study design was based on self-reported experiences, which might be influenced by social desirability or recall bias. However, thematic saturation and purposive sampling facilitated to ensure a complete exploration of participant perspectives. As participation was voluntary and based on purposive sampling, the possibility of selection bias cannot be excluded, particularly as students who were more interested in sleep health may have been more likely to participate. Information regarding prior interest in sleep medicine or related health topics was not formally assessed, and therefore its influence on participant responses cannot be excluded. As the study relied on self-reported narratives, the possibility of social desirability bias cannot be excluded, particularly among medical students who may underreport symptoms or overemphasize self-management behaviours. Further research might explore awareness about sleep health across multiple medical colleges and analyse interventions targeted to enhance sleep recognition disorders among medical trainees.

## 5 Conclusion

These findings underscore the urgent need for detailed sleep health education as a part of medical education. Furthermore, there is a threatening paradox in that, despite their theoretical access to knowledge, medical students often fail to apply it practically to their own wellbeing. This existing gap between knowledge and behaviour emphasizes the necessity of strengthening perception of risk, recognition of symptoms, and awareness about help-seeking pathways within medical curriculum.

Medical colleges might therefore opt for strengthening student support systems and improve open dialogue around sleep health to enhance timely recognition of symptoms-related to sleep and promote appropriate help-seeking behaviour.

In conclusion, this study reveals an alarming mismatch between awareness of OSA and self-experience with symptoms and help-seeking behaviour among medical students. Hence, medical education must go beyond knowledge from textbooks and actively engage students in preventive, reflective and health-promoting activities. Recognizing OSA as an important personal health issue might denote an essential step in encouraging future healthcare professional to give importance for their own sleep health and involve in timely help-seeking behaviour.

## Data Availability

The raw data supporting the conclusions of this article will be made available by the authors, without undue reservation.
