# Supplementary material for: Perceived risk, symptoms and help-seeking behaviour for obstructive sleep apnoea among undergraduate medical students: a qualitative study
Source: Front Public Health. 2026 Jun 25;14:1857585. doi: 10.3389/fpubh.2026.1857585 (PMC13346073; doi:10.3389/fpubh.2026.1857585)
Supplement: Supplementary file 1 [file Data_Sheet_1.PDF]

<u>Semi-Structured Interview Guide</u>

Section A: Sleep Patterns and Daily Routine

1. Can you describe your usual sleep routine during a typical week?
2. What factors usually help or disturb your sleep?
3. How have your sleep patterns changed since joining medical college?
4. How does sleep (or lack of sleep) affect your academic and personal life?
5. Have you ever tried to improve your sleep quality? If yes, how?

Section B: Academic Stress and Lifestyle

1. What are the common sources of stress in your routine as a medical student?
2. How do academic demands affect your sleep and daily lifestyle?
3. What influences your choices regarding food, exercise, and sleep?
4. Have you made any lifestyle changes since joining medical college? What prompted them?

Section C: Awareness and Perceived Risk of Obstructive Sleep Apnoea

1. Have you ever noticed or been told about snoring, pauses in breathing, or restlessness during sleep?
2. What do you know about obstructive sleep apnoea?
3. Do you think you are personally at risk of OSA? Why or why not?
4. How often do you experience excessive daytime sleepiness or fatigue, and what do you attribute it to?
5. Have you experienced weight changes or fatigue that you feel may be related to sleep problems?

Section D: Help-Seeking Behaviour and Coping

1. What are your thoughts on seeking help for sleep-related issues?
2. Have you ever considered seeking professional help for sleep or stress-related concerns? Why or why not?
3. What strategies do you usually use to manage stress or sleep disturbances?
