# Supplementary material for: Perceived risk, symptoms and help-seeking behaviour for obstructive sleep apnoea among undergraduate medical students: a qualitative study
Source: Front Public Health. 2026 Jun 25;14:1857585. doi: 10.3389/fpubh.2026.1857585 (PMC13346073; doi:10.3389/fpubh.2026.1857585)
Supplement: Supplementary file 2 [file Data_Sheet_2.PDF]

<u>Coding and Theme Development Process</u>

**Raw Participant Statement**	**Initial Code**	**Developed Category**	**Final Theme**
"I do not think I am at risk."	Low perceived risk	Underestimation of susceptibility	Misconceptions and limited awareness about OSA
"Every day I feel sleepy because of irregular sleep habits."	Daytime sleepiness attributed to lifestyle	Normalization of symptoms	Symptom recognition and normalization
"I am changing my sleep schedule and it's working."	Self-management approach	Preference for self-care	Barriers to help-seeking
"Being a medical student, sleeping correctly sounds impossible."	Academic burden affecting sleep	Impact of medical training	Impact of medical school lifestyle

Codes were generated inductively from participant narratives. Similar codes were grouped into broader conceptual categories, which were subsequently refined into final themes through iterative discussion among the research team.
